# A systems biology analysis of the changes in gene expression via silencing of HPV-18 E1 expression in HeLa cells

**DOI:** 10.1098/rsob.130119

**Published:** 2014-10-08

**Authors:** Andres Castillo, Lu Wang, Chihaya Koriyama, Yoshito Eizuru, King Jordan, Suminori Akiba

**Affiliations:** 1 Department of Physiology and The Basic Sciences School, Health Faculty at Universidad del Valle, Cali, Colombia; 2 UniValle-Georgia Tech Genome Research Center, Health Faculty at Universidad del Valle, Cali, Colombia; 3 School of Biology, Georgia Institute of Technology, Atlanta, GA, USA; 4 Department of Epidemiology and Preventive Medicine, Kagoshima University Graduate School of Medical and Dental Sciences, 8-35-1 Sakuragaoka, Kagoshima 890-8544, Japan; 5 Division of Oncogenic and Persistent Viruses, Center for Chronic Viral Diseases, Kagoshima University Graduate School of Medical and Dental Sciences, 8-35-1 Sakuragaoka, Kagoshima 890-8544, Japan; 6 PanAmerican Bioinformatics Institute, Santa Marta, Magdalena, Colombia

**Keywords:** human papillomavirus, HPV E1 protein, siRNAs, HeLa cells, microarrays analysis, EP300 protein

## Abstract

Previous studies have reported the detection of a truncated E1 mRNA generated from HPV-18 in HeLa cells. Although it is unclear whether a truncated E1 protein could function as a replicative helicase for viral replication, it would still retain binding sites for potential interactions with different host cell proteins. Furthermore, in this study, we found evidence in support of expression of full-length HPV-18 E1 mRNA in HeLa cells. To determine whether interactions between E1 and cellular proteins play an important role in cellular processes other than viral replication, genome-wide expression profiles of HPV-18 positive HeLa cells were compared before and after the siRNA knockdown of E1 expression. Differential expression and gene set enrichment analysis uncovered four functionally related sets of genes implicated in host defence mechanisms against viral infection. These included the toll-like receptor, interferon and apoptosis pathways, along with the antiviral interferon-stimulated gene set. In addition, we found that the transcriptional coactivator E1A-binding protein p300 (EP300) was downregulated, which is interesting given that EP300 is thought to be required for the transcription of HPV-18 genes in HeLa cells. The observed changes in gene expression produced via the silencing of HPV-18 E1 expression in HeLa cells indicate that in addition to its well-known role in viral replication, the E1 protein may also play an important role in mitigating the host's ability to defend against viral infection.

## 2. Introduction

The International Agency for Research on Cancer has indicated that there is convincing evidence that infection with human papilloma virus type 16 (HPV-16) and HPV-18 can result in cervical cancer, as well as in cancers of the vulva, vagina, penis and anus [1]. HPV DNA has also been found in cancers of the oral cavity, oropharynx, larynx, oesophagus and lung, and thus its association with cancers of the upper aero-digestive tract has also been suspected [2–5]. In addition, HPV DNA has been detected in cell lines derived from cervical cancers: HPV-16 DNA in the cell lines CaSki and SiHa, and HPV-18 DNA in HeLa cells [6–9]. Owing to the difficulty of establishing tissue culture systems, which are susceptible to transformation by HPV, these cell lines provide unique systems to study the expression of HPV-16 and HPV-18 genes in cells derived from human tumours.

In HeLa cells, a cervical adenocarcinoma-derived cell line containing multiple copies of integrated HPV-18 DNA [10–14], HPV-18 early mRNAs are transcribed as polycistronic RNA [10], which give rise to three differentially spliced mRNA species [11,12]. These early mRNAs contain information for the translation of three potential HPV-18 proteins: E1, E6 and E7. Seedorf *et al*. [11] detected two early proteins—E7 (12 kDa) and a truncated E1 (70 kDa)—in HeLa cells, as assessed using polyacrylamide gels, following hybrid selection and Western blotting analyses, in which the sizes were consistent with the sizes predicted from the HPV-18 DNA sequence.

Abundant information is available on the E7 protein and its role in both the virus infection cycle and carcinogenesis. The viral protein E7 can induce cell immortalization by binding to pRb, a tumour suppressor protein, which binds to and inactivates the E2F transcription factor. E2F is released from pRb, which results in the transcription of genes involved in DNA replication and cell division [15]. In addition, E1 is a replicative helicase protein, which binds to the host DNA polymerase alpha-primase [16], replication protein A [17] and topoisomerase I to perform viral replication [18]. Moreover, E1 also interacts with the molecular chaperones, Hsp40 and Hsp70 [19], cellular WD-repeat protein 80, which maintains the viral genome in keratinocytes [20], E/cdk2 cyclin complex [21], and with cellular proteins involved in chromatin remodelling and co-transcription activation, such as histone H1 [22] and Ini1/hSNF5, a subunit of the SWI/SNF chromatin remodelling complex [23]. However, it is still unknown whether these interactions play a role in cellular processes other than viral replication.

To determine if these potential interactions play an important role in cellular processes other than viral replication, HPV-18 *E1* mRNA, which is endogenously expressed in HeLa cells, was silenced using short interfering RNA sequences (siRNAs). Subsequently, we examined changes in the expression of cellular genes using microarray analysis. Differentially expressed genes were analysed using gene set enrichment in order to look for functionally related sets of genes that may be coordinately changed upon the *E1* mRNA knockdown.

## 3. Material and methods

### 3.1. Cell lines

Both HeLa cells (ATCC: CCL-2), which are HPV-18-positive human cervical adenocarcinoma cells, and A549 cells (ATCC: CCL-185), which are HPV-negative human lung adenocarcinoma cells, were maintained in minimal essential medium (Eagle's MEM, Nissui Pharmaceutical Co., Tokyo, Japan), supplemented with L (+)-glutamine (Wako, Japan) and 10% fetal bovine serum.

### 3.2. Short interfering RNA sequences

Six siRNAs targeting HPV-18 *E1* mRNAs (siE1.1–6) were selected using an algorithm based on guidelines developed by Ui-Tei *et al*. [24] and the web-based online software system sidirect [25]. The siRNA molecule sequences were designed under the following conditions: A/U at the 5′ end of the antisense strand; G/C at the 5′ end of the sense strand; at least five A/U residues in one-third from the 5′ end of the antisense strand; and the absence of more than nine GC alignments (table 1). These six siRNAs and non-targeting control siRNA (siControl: 5′-UAG CGA CUA AAC ACA UCA A-3′) were synthesized by Sigma GenosyssiRNA service (Sigma-Aldrich Japan K.K., Tokyo, Japan).

**Table 1.** Short interfering RNA (siRNA) sequences targeting HPV-18 E1 mRNA expression in HeLa Cells.

ID	sequences	HPV-18 genome nucleotide position	mismatch tolerance
siRNA E1.1	5′-GUA ACG GCU GGU UUU AUG UAC-3’ 5’-ACA UAA AAC CAG CCG UUA CAA-3’	850–870	3
siRNA E1.2	5’-GUU AAG UCC ACG GUU ACA AGA-3’ 5’-ACA UAA AAC CAG CCG UUA CAA-3’	1133–1153	3
siRNA E1.3	5’-GUA CCA UAG CAC AAU UAA AAG-3’ 5’-UUU AAU UGU GCU AUG GUA CAU-3’	1396–1416	3
siRNA E1.4	5’-CGA UAU GGC AUU UGA AUA UGC-3’ 5’-AUA UUC AAA UGC CAU AUC GCU-3’	1949–1969	3
siRNA E1.5	5’-CCU GCG AUA CCA ACA AAU AGA-3’ 5’-UAU UUG UUG GUA UCG CAG GAA-3’	2166–2186	3
siRNA E1.6	5’-GAA UUC ACA UAG UCA UUU UUG-3’ 5’-AAA AUG ACU AGU GGA AUU CAG-3’	2336–2356	3
E1 primers	5’-TGG CTG ATC CAG AAG GTA CA-3’ 5’-CGG TTC CAA CCA AAA ATG AC-3’	811–831 2366–2347	

### 3.3. Transfection of short interfering RNA

Short interfering RNA was delivered to HeLa or A549 cells using Oligofectamine transfection reagent (Invitrogen), according to the manufacturer's instructions. Briefly, HeLa or A549 cells were seeded at 10^4^ cells per well in a 6-well plate and cultured for 24 h at 30–50% confluence. For transfection, 4 µl of Oligofectamine and 100 nM siRNA were diluted in Opti-MEM I reduced-serum medium (Invitrogen) in a final volume of 200 µl. Cultured cells in 0.8 ml Opti-MEM I were transfected with a mixed solution of siRNA, and 4 h later the culture was supplemented with 0.5 ml MEM and l(+)-glutamine containing 30% fetal bovine serum. The mRNA expression levels were examined after 48 h of transfection. The status of cells was quantified using trypan blue stain (Gibco BRL) after 48, 72 and 96 h of transfection. The siRNA transfections were repeated after 72 h with changing media. The number of cells for each well was quantified three times using a haemocytometer (Burker-Turk deep 1/10 mm; Erma, Tokyo, Japan).

### 3.4. Quantitative real-time RT-PCR

Total RNA was extracted using an Isogen-LS RNA extraction kit (Nippon Gene, Tokyo, Japan). Genomic DNA was removed from RNA preparations using the DNase I recombinant RNase-free (Roche, Germany) prior to RT-PCR. After quantification of RNAs using the NanoDrop ND-1000 Spectrophotometer (NanoDrop Technologies), RNA samples were stored at −20°C, until needed for no more than 2 days. Single-strand cDNAs were synthesized from mRNAs and quantified using the QuantiTectTM SYBR Green RT-PCR Kit (Qiagen) and ABI Prism 7000 sequence detection system (Applied Biosystems). Glyceraldehyde 3-phosphate dehydrogenase (*GAPDH*) gene was used as an internal control to normalize the cDNA expression levels. *GAPDH* primer sequences for forward and reverse were 5′-TGA TGA CAT CAA GAA GGT GGT GAA G-3′ and 5′-TCC TTG GAG GCC ATG TGG GCC AT-3′, respectively. For each target gene, 1 µl of cDNA was amplified in a total volume of 20 µl containing 2 × QuantiTect SYBR Green RT-PCR master mixes supplemented with 300 nM of each primer. The data were analysed using ABIprism 7000 SDS software (Applied Biosystems). For all the samples, crossing points (CP) normalized expression of the target gene versus *GAPDH* for each treatment or control sample were calculated [26] using the following formula: 2^−(ΔCP treatment sample^^–^^ΔCP control sample)^. Each sample was assayed three times.

### 3.5. RNA-seq analysis

RNA-seq data from a transcriptome analysis of HPV-18+ HeLa cells [27] were downloaded from the European Nucleotide Archive (ENA; accession ERP000959). Sequence reads from this dataset were mapped to the HPV-18 complete genome reference sequence (NCBI Refseq accession NC_001357) using the program Bowtie2 [28]. HPV-18 gene expression levels were quantified with log_10_-transformed sequence coverage values (i.e. number of mapped tags per position), and mapped reads were visualized along the HPV-18 genome sequence using the Integrative Genomics Viewer [29,30].

### 3.6. Microarray analysis

RNA qualities were examined by capillary electrophoresis using the Agilent 2100 Bioanalyzer (Agilent Technologies, Santa Clara, CA, USA), and their qualities were confirmed (28S/18S rRNA, E260/E280 ratio > 1.8). Microarray analysis (including labelling, hybridization, image scanning and sample conditions) was performed by Takara Bio Dragon Genomix Center (Mie, Japan). Briefly, fluorescent-labelled cDNAs were generated from 200 ng of total RNA in each reaction using the Agilent Quick Amp labelling kit in two colours. The cyanine 5-labelled cDNAs from the siE1.6-treated HeLa cells were mixed with the same amount of reverse-colour cyanine 3-labelled cDNAs from the non-treated control cells and then applied to a whole human genome oligonucleotide microarray Kit (Agilent Technologies), which contained 41 000 unique human genes and transcripts. These transcripts all contained public domain annotations (http://www.chem.agilent.com/CAG/bsp/gene_lists.asp).

Upon hybridization and washing, the Agilent dual-laser DNA microarray scanner scanned the arrays. The data were then extracted from images using the Agilent Feature Extraction v. 9.5 software, and candidate genes were selected using genespringgx software (Agilent Technologies). An intensity-dependent normalization (LOWESS: locally weighted regression) was applied to correct artefacts caused by nonlinear rates of dye incorporation as well as inconsistencies of the relative fluorescence intensity between the red and green dyes. Differentially expressed genes were then identified by comparing the log_2_ ratio of expression levels between conditions with the overall log_2_ expression signals (LogRatio versus Log Processed Signal; electronic supplementary material, figure S1).

### 3.7. Gene set enrichment analysis

The potential functional significance of the differentially expressed genes was analysed using gene set enrichment analysis by combining the Ingenuity Pathway Analysis tool (http://www.ingenuity.com/products/ipa) with analyses of custom gene sets taken from the literature (electronic supplementary material, figure S2) [31]. To do this, the enrichment of differentially expressed genes in pathways, or functionally coherent sets of genes, was determined using the Fisher's exact test with the *p*-value determined as
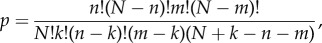
where *N* is the total number of genes on the microarray, *n* is the total number of differentially expressed genes, *m* is the total number of genes in the pathway and *k* is the total number of differentially expressed genes in the pathway.

### 3.8. Validation of microarray data

Validation of the microarray data was performed using a real-time RT-PCR assay for *EP300* and cyclin-dependent kinase inhibitor 1A (*CDKN1A*) mRNAs using real-time RT-PCR. The primer sequences for *EP300* forward and reverse were 5′-CTC CAA CTT TCT GCC ACA ACA-3′ and 5′-CCA GAA TCC CTT CCC TTT CG-3′, respectively. For *CDKN1A*, the primer sequences forward and reverse were 5′-GGA CCT GGA GAC TCT CA-3 and 5′-CCT CTT GGA GAA GAT CAG-3, respectively. The transcript level of each gene was determined using the QuantiTectTM SYBR Green RT-PCR Kit (Qiagen) and ABIprism 7000 SDS. *GAPDH* mRNA was also used as an internal control to normalize the mRNA expression levels.

## 4. Results

### 4.1. Expression of a full-length E1 mRNA and protein

Previous reports have shown evidence suggesting that the HPV-18 early genes E6, E7 and E1 are transcribed as a polycistron that terminates within the E1 ORF [10,12]. This has been taken to indicate the production of a truncated E1 protein with attenuated viral replication activity [14]. However, Western blot analysis of HeLa cell protein extracts revealed a 70 kDa E1 protein [11], which is consistent with the molecular weight calculated from the full-length E1 protein sequence (73.75 kDa) and substantially larger than the calculated molecular weight of the protein corresponding to the shorter E1 transcript (58.55 kDa). To address this apparent contradiction, we re-evaluated HPV-18 gene expression patterns using RNA-seq data from a more recently published transcriptome analysis of HeLa cells [27]. These RNA-seq data support the expression of a full-length E1 mRNA transcript, with the caveat that there may indeed be a higher-abundance E6–E7–E1 polycistron that contains a shorter E1 sequence (electronic supplementary material, figure S3). This shorter E1 sequence may be responsible for the previously observed sequestration of cofactors needed to initiate HPV-18 replication in HeLa cells [14]. Nevertheless, in light of our new results on E1 mRNA expression and the previous results on E1 protein expression [11], we conclude that HeLa cells are in fact capable of producing full-length E1 protein product.

### 4.2. Both siE1.2 and siE1.6 molecules specifically silenced HPV-18 E1 endogenous mRNA expression in HeLa cells

**Figure 1.**
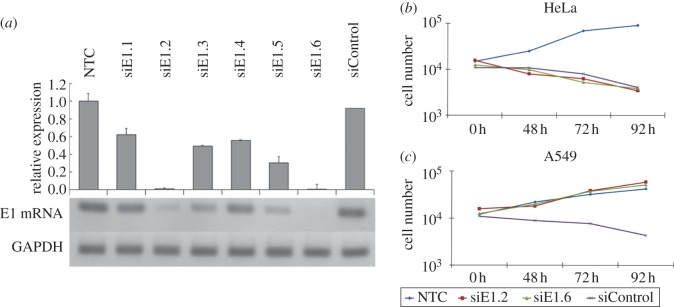
Effects of siRNA molecules on endogenous HPV18-E1 expression and HeLa cell numbers. (*a*) Six siRNAs targeted to endogenous HPV18-E1 mRNA (siE1.1, siE1.2, siE1.3, siE1.4, siE1.5 and siE1.6) were transfected at 100 nM for 48 h. The mRNAs were synthesized from single-stranded cDNAs and quantified using the QuantiTectTM SYBR Green RT-PCR Kit (Qiagen) and ABIprism 7000 sequence detection system (Applied Biosystems) according to the manufacturers' instructions. GAPDH was used as an internal control to normalize the cDNAs expression levels. siRNA siE1.2 and siE1.6 molecules showed strong and specific silencing of endogenous HPV18-E1 expression in HeLa cells. In addition, the siControl did not show any effect on endogenous E1 expression. (*b*) In HeLa cells, the cell number was decreased after transfection of siRNA siE1.2 and siE1.6 compared with HeLa cells without siRNA transfection. (*c*) In HPV-negative A549 cells, the cell number did not show any effect after siRNA transfections. Furthermore, the siControl showed a negative effect in the cell numbers in both cell lines. The cell number was quantified using trypan blue stain (Gibco BRL) after 48, 72 and 96 h. The number of cells in each well was quantified three times using a haemocytometer (Burker-Turk deep 1/10 mm; Erma, Tokyo, Japan). NTC, non-treatment controls.

**Figure 2.**
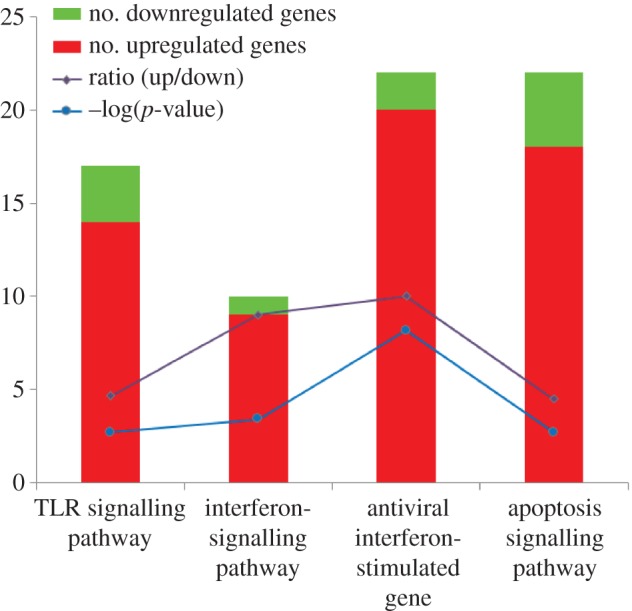
Enrichment of differentially expressed genes in four closely related gene sets, including three canonical pathways and one functional group: TLR signalling pathway, IFN signalling pathway, antiviral ISG and apoptosis pathway. The numbers of genes upregulated and downregulated in E1-silenced HeLa cells are shown on the same scale as the ration of upregulated/downregulated genes and the log-normalized *p*-values for the gene set enrichment analysis.

First, HPV-18 *E1* mRNA expression in HeLa cells was quantified using real-time RT-PCR (figure 1*a*). *GAPDH* expression was used as an internal control to normalize *E1* mRNA expression. Next, *E1* mRNA expression was quantified after 48 h of transfection for each siRNA. As shown in figure 2*a*, *E1* mRNA expression was strongly suppressed by siE1.2 and siE1.6 molecules, and the effects of siE1.1, siE1.3, siE1.4 and siE1.5 molecules were moderate in HeLa cells. Transfection of the siControl molecule did not affect the *E1* mRNA expression. In addition, the number of HeLa cells was decreased after transfection of siE1.2 and siE1.6 molecules compared with the non-treated control (cells without siRNA transfection) (figure 1*b*). By contrast, the number of A549 cells was not decreased after transfection of siE1.2 and siE1.6 siRNA molecules (figure 1*c*). Transfection of the siControl molecule decreased the cell number in both cell lines. Taken together, these results suggested that transfection of both siE1.2 and siE1.6 molecules could silence *E1* mRNA expression in HeLa cells and decrease the cell number by one-tenth.

### 4.3. Expression data analysis identified 2669 differentially expressed cellular genes after silencing E1 endogenous mRNAs in HeLa cells

**Figure 3.**
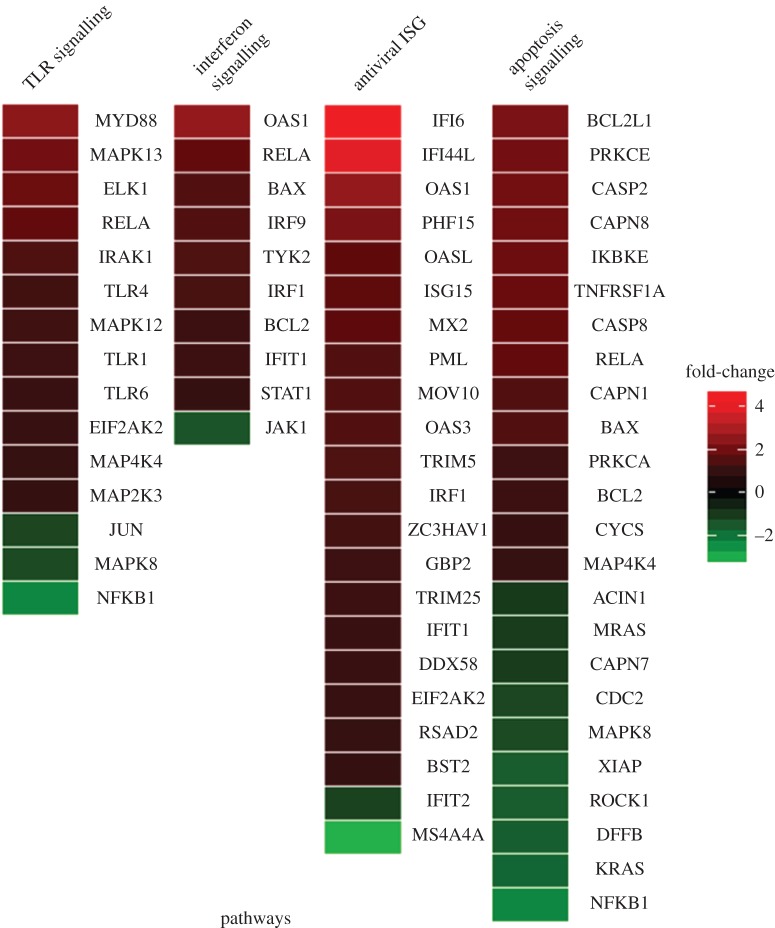
Differentially expressed genes in the four enriched gene sets following E1 silencing. Log_2_ gene expression fold change values between conditions are coloured as shown in the key.

The cellular gene expression profile of HeLa cells was compared before and after siE1.6 transfection using the Agilent human oligonucleotide microarray analysis, which contained 41 000 unique human gene transcripts. The microarray data were normalized and the data quality was assessed. Globally, 2669 differentially expressed genes with log_2_ ≥ 1 were identified, comprising 1718 upregulated and 951 downregulated genes. The potential biological significance of these differentially expressed genes was evaluated using gene set enrichment analysis. Upon HPV-18 E1 silencing, we found significant enrichment (*p* < 0.05, Fisher's exact test) of differentially expressed genes in four closely related gene sets, including three canonical pathways and one functional group: the toll-like receptor (TLR) signalling pathway, the interferon (IFN) signalling pathway, the antiviral interferon-stimulated genes (ISG) and the apoptosis pathway (figure 2 and table 2). In addition, the majority of genes in these sets were upregulated after E1 silencing (figures 2 and 3).

**Table 2.** Functional role and statistics on differentially expressed genes of specific pathways and gene sets.

pathway/set	description	no. genes in set	no. DE genes in set	upregulated gene	downregulated gene	*p*-value (Fisher's exact test)
TLR signalling pathway	signal transduction process characterized by specific families of PRR which are responsible for detecting microbial pathogens and generating innate immune responses	57	17	14	3	0.002
IFN signalling pathway	immune responses pathway in which IFN initiates immune responses via signal transduction sub-pathways, resulting in the activation of downstream gene expression	34	10	9	1	0.0004
antiviral ISG	set of ISG characterized with antiviral function	42	22	20	2	7 × 10^−9^
apoptotic signalling	a coordinated, energy-dependent signal transduction process that involves the activation of caspases and a cascade of events that link the initiating stimuli to programmed cell death	92	24	14	10	0.002

**Figure 4.**
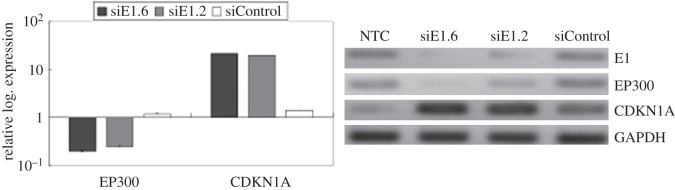
*EP300* downregulation after silencing of HPV-18 E1. Confirmation of micro-array data using real-time RT-PCR. Transcript levels of *EP300* and *CDKN1A* were quantified using RT-PCR. *EP300* and *CDKN1A* gene expressions were affected after siE1.6 and siE1.2 transfections, at 100 nM for 48 h, but not after siControl transfection. Decreased *EP300* and increased *CDKN1A* expression were consistent with the results obtained from the microarray analysis. The relative logarithmic expressions were normalized against the non-treatment control (NTC). *GAPDH* was used as a reference gene control.

Our results also showed a significant suppression of *EP300* expression (−2.20-fold change). Furthermore, a set of cyclin genes, such as *E2* (*CCNE2*), *A2* (*CCNA2*) and *B2* (*CCNB2*), showed lower expression. Other suppressed genes relating to the cell cycle include retinoblastoma 1 (*RB1*), cyclin-dependent kinase inhibitor 1B (*CDKN1B*), Polo-like kinase 1 (*PLK1*), cell division cycle 25 homologue C (*CDC25C*), cell division cycle 2 (*CDC2*), and SMAD family members 2 (*SMAD2*) and 4 (*SMAD4*)*.* By contrast, the *CDKN1A* gene showed a higher score of overexpression (2.14-fold change). The cyclin D3 (*CCND3*), tyrosine 3-monooxygenase/tryptophan 5-monooxygenase-activation proteins, beta polypeptide (YWHAB) and cell division cycle 25 homologue B (*CDC25B*) were also overexpressed. The microarray results of *EP300* and *CDKN1A* expressions were validated using real-time RT-PCR. Transfection of siE1.6 molecules decreased *EP300* and increased *CDKN1A* expression, which was consistent with the results of the microarray analysis (figure 4). Furthermore, similar results were obtained using siE1.2 molecule transfection.

## 5. Discussion

This study revealed that silencing of the HPV-18 *E1* endogenous mRNA expressions in HeLa by siRNA molecules induced changes in the expression of cellular genes, specifically with high expression in three canonical pathways and one functional group: the TLR signalling pathway, the IFN signalling pathway, the antiviral ISG and the apoptotic pathway (figures 2 and 3).

Targeting the TLR signalling pathway in elucidating the cellular and molecular mechanisms of cervical cancer has gained tremendous importance [32]. TLR-4 was overexpressed in cervix cancer, and its activation by LPS promotes proliferation and anti-apoptosis in HeLa cells *in vitro* [33]. However, members of the interferon regulatory factor (IRF) family of DNA-binding transcription factors have played a role in growth regulation, antiviral responses and transcriptional induction of IFN-activated early response genes [34] and critical mediators of the induction of early viral transcription and replication in several mucosal HPVs, which require IRF-1 binding to a conserved interferon response element [35]. HPV infections disrupt cytokine expression and signalling with E6 and E7 oncoproteins, and particularly target the type I IFN pathway [36]. However, the precise mechanism in which the IFN complex acts on tumours remains unknown, although its use in clinical practice has been widely recommended, particularly in tumours that are resistant to conventional treatments [37]. The TLRs belong to the pathogen-associated pattern recognition receptor (PRR) family. Upon viral infection, TLRs can sense specific molecular patterns in viruses and activate IRFs, resulting in the transcriptional induction of IFN. Activated by the TLR signalling pathway, IFNs signal via the JAK/STAT pathway to induce the production of ISGs. Activation of the IFN signalling pathway directly results in the production of ISGs. Several ISGs function to block virus replication, such as IFIT1. Other upregulated ISGs, such as EIF2AK2, which encodes the dsRNA-dependent protein kinase R, can initiate an additional antiviral response in the host cell, which eventually results in apoptosis. Cell death is an essential component of the host cell immune machinery. It can be induced by specific ISGs. Our expression data indicated that the apoptotic pathway might be suppressed prior to silencing of the viral E1 protein.

How can HPV-18 E1 expression affect a large number of genes? Whereas HeLa cells are capable of producing full-length E1 proteins, the dominant product may be the previously observed *E6–E7–E1* polycistron that encodes a truncated E1 protein (electronic supplementary material, figure S3). This shorter HeLa cell-derived HPV-18 E1 protein is truncated at the carboxyl terminus, but still retains carboxyl terminal protein-binding domains that can associate with host cellular factors such as cyclin E/CDK2 [14] and Ini1/hSNF5, a subunit of the SWI/SNF chromatin remodelling complex [23]. Recruitment of the SWI/SNF complex by E1 can alter the local arrangement of transcription factors, replication factors and histones. This rearrangement may reprogram the transcriptional and replication state of DNA. We also found a downregulation of the transcription and translation of EP300 (figure 4), a histone acetyltransferase that epigenetically regulates chromatin remodelling via histone acetylation, which enables access to several transcription factors, thereby activating gene expression [38]. However, the regulatory mechanism of *EP300* expression is not well understood. According to a report by Yu *et al*. [39], early growth response 1 (Egr-1), which binds to GC-rich elements in the promoters, acts upstream of p300/CBP. Egr-1, a zinc finger transcriptional factor, accumulates in the cell nucleus upon stimulation by mitogens and a variety of cytokines, as well as extracellular effects, including hypoxia and 17-β-oestradiol [40,41]. Recently, Saegusa *et al*. [42] reported that *EP300* gene expression is upregulated by Egr-1 binding to the promoter region of the *EP300* gene in endometrial carcinoma cells. Although there was no evidence of Egr-1 regulation by HPV-encoded proteins, the mRNA levels of *Egr-1* in cervical cancer were significantly higher compared with normal tissue [43]. In this study, we observed a decrease of *Egr-1* expression after the silencing of HPV-18 *E1* mRNA, suggesting that *Egr-1* can be stimulated by E1. However, negative feedback of EP300 to Egr-1 expression was suggested [39–42]. Moreover, Bouallaga *et al*. [44] reported that EP300 is recruited by the HPV-18 enhanceosome and is required for HPV18 transcription in HeLa cells. Also, the cell confluence ceiling during the siRNA experiments prior to harvesting was at least 50%, and in control cultures was almost 90%. The above is important because it proves that change in the expression of cell cycle genes such as RB1, CDC25C, CDC2 and CDKN1A has the consequence that cell proliferation is arrested.

An important question is whether the siRNA targeting of E1 also affects the stability (and thus expression) of the *E1–E6–E7* polycistronic mRNAs. To address this point, we conducted two additional qPCR analyses. We carefully evaluated both *E6* and *E7* mRNA expression after siRNA treatments in HeLa cells, and we did not find any significant effect on the expression of *E6* and *E7* after treatment with siRNAs. Our explanation for these results is based on the fact that the process of maturation of *E1–E6–E7* polycistronic mRNAs occurs in the nucleus, and then each one of them (*E1*, *E6* and *E7* mRNAs) are exported to the cytosol. On the other hand, many studies have shown that the process of siRNA inhibition is performed mainly in the cytosol. Therefore, we consider there to be little chance that the siRNA targeting of *E1* can affect the stability and expression of the *E1–E6–E7* polycistronic mRNAs, since they may be in different places in the cell.

## 6. Conclusion

**Figure 5.**
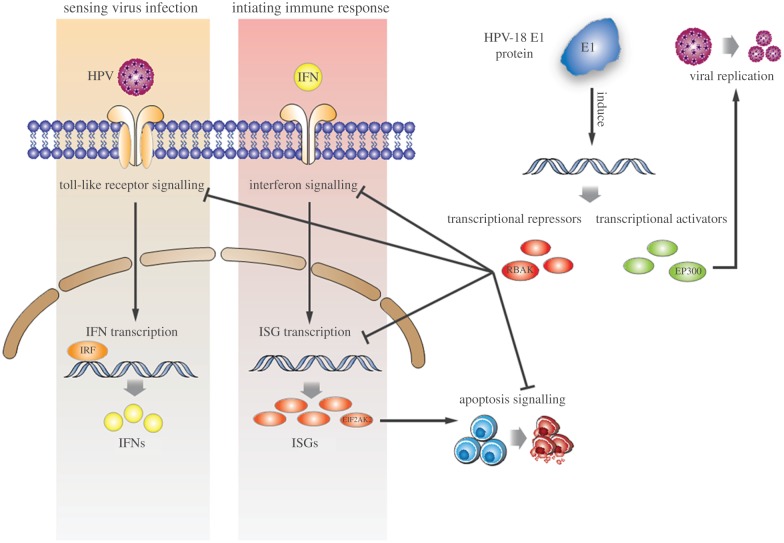
Schematic of the mechanisms by which the viral E1 protein may mitigate the host's ability to defend against viral infection. E1 protein expression induces transcriptional repressors that coordinately downregulate the activity of pathways involved in both the sensing of viral infection and the initiation of immune responses to infection, including apoptosis. E1 protein expression induces transcriptional activators involved in viral replication.

Taken together, our data indicate a consistent suppression pattern at the transcriptional level induced by the HPV-18 E1 protein across four intensively collaborating components of the host cell immune machinery (figure 5), suggesting that in addition to its well-understood role in viral replication, the viral E1 protein may also play an important role in mitigating the host's ability to defend against infection.

## Supplementary Material

Supplementary Information
