# Supplementary material for: A systems biology analysis of the changes in gene expression via silencing of HPV-18 E1 expression in HeLa cells
Source: Open Biol. 2014 Oct 8;4(10):130119. doi: 10.1098/rsob.130119 (PMC4221889; doi:10.1098/rsob.130119)
Supplement: Supplementary Information [file rsob130119supp1.docx]

Supplementary Information for: **A systems biology analysis of the changes in gene expression via silencing of HPV-18 E1 expression in HeLa cells** Andres Castillo, Lu Wang, Chihaya Koriyama, Yoshito Eizuru, I. King Jordan, Suminori Akiba


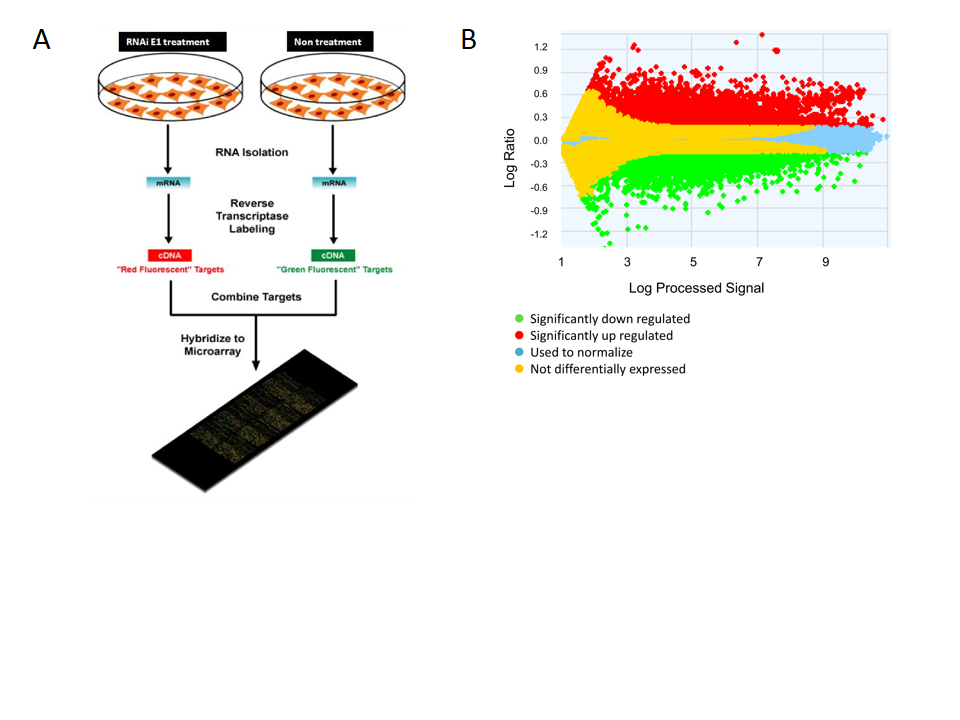


Supplementary Figure 1. **Microarray analysis of gene expression in RNAi E1 treated and non-treated HeLa cells**. A) Experimental workflow of the microarray analysis. mRNA from both RNAi E1 treated and non-treated HeLa cells are isolated and converted to cDNA library with red and green fluorescent labels, respectively. Combined targets are then applied to Whole Human Genome Oligo Microarray Kit. B) Scatter plot of the Log processed signal and the Log signal ratio for each probe.


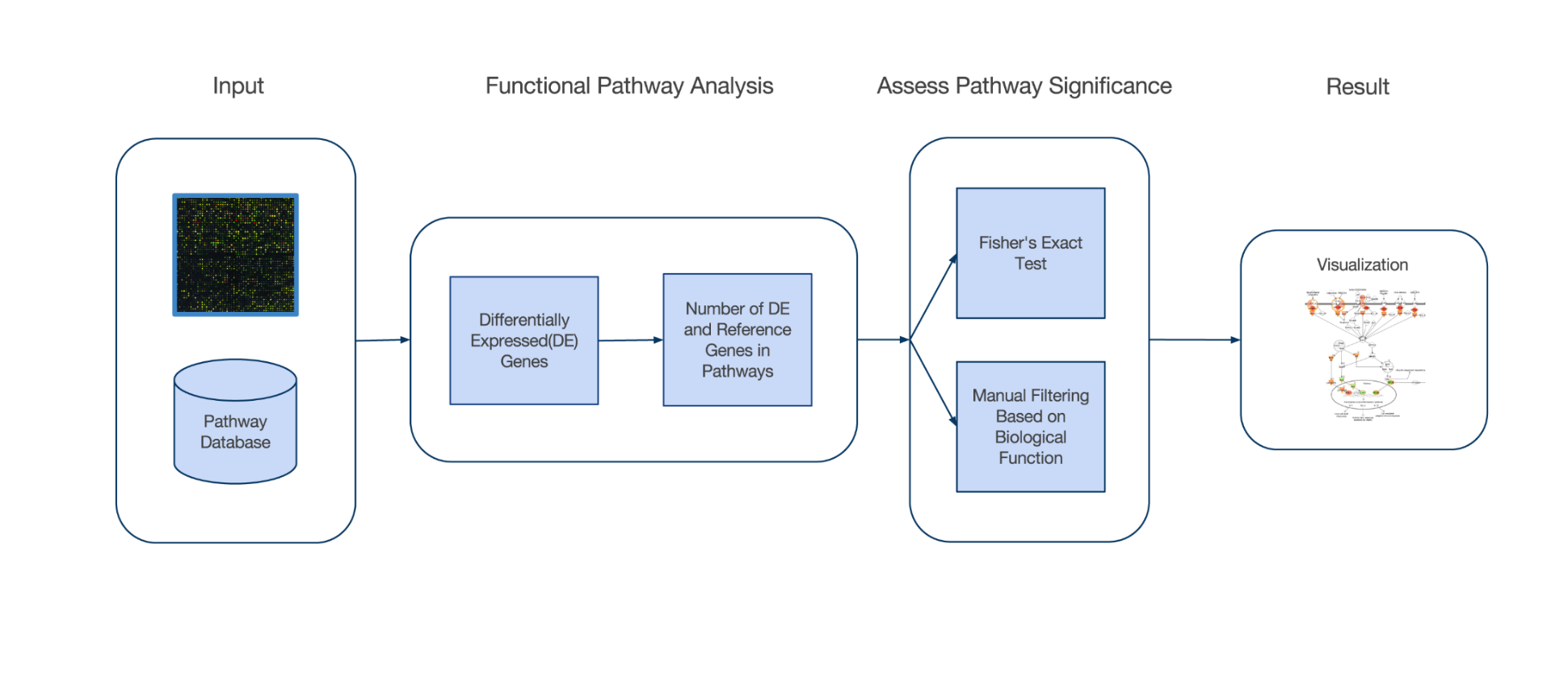


A


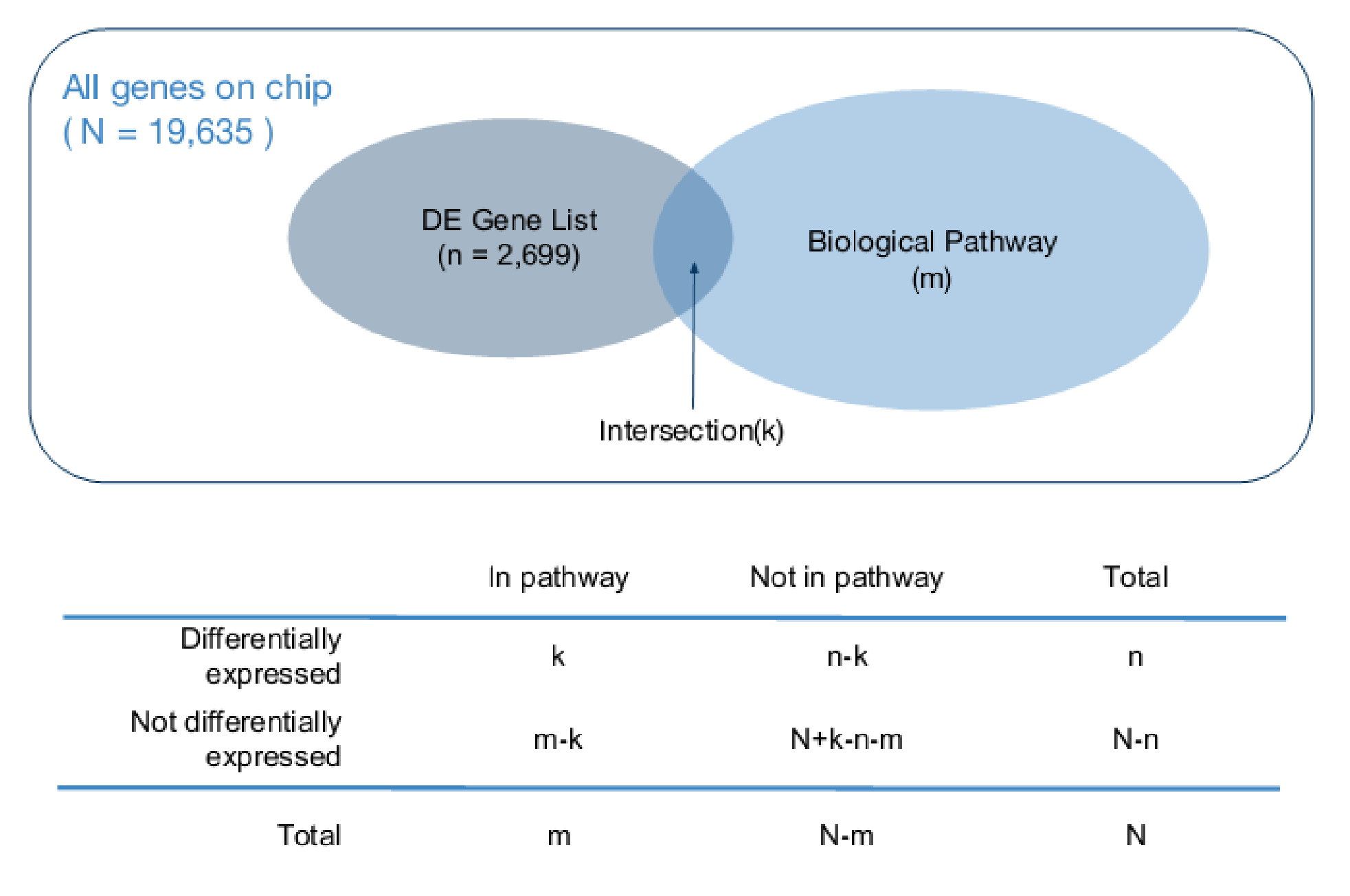


B

Supplementary Figure 2. **The workflow of the Gene Set Enrichment Analysis (GSEA).** A) Expression data from microarray were taken together and differentially expressed genes are identified and compared against canonical pathways and known functional gene sets. For each pathway, number of differentially expressed(DE) genes and reference genes are counted and compared. Pathways and functional gene sets with significant enrichment of DE genes were manually filtered based on biological function, resulting in functionally coherent gene sets with significant enrichment of DE genes. B) Two by two contingency table for Fisher’s Exact Test in which *N* is the total number of genes on chip, *n* is the total number of DE genes, m is total number of gene in pathway and k is the total number of DE gene in pathway.


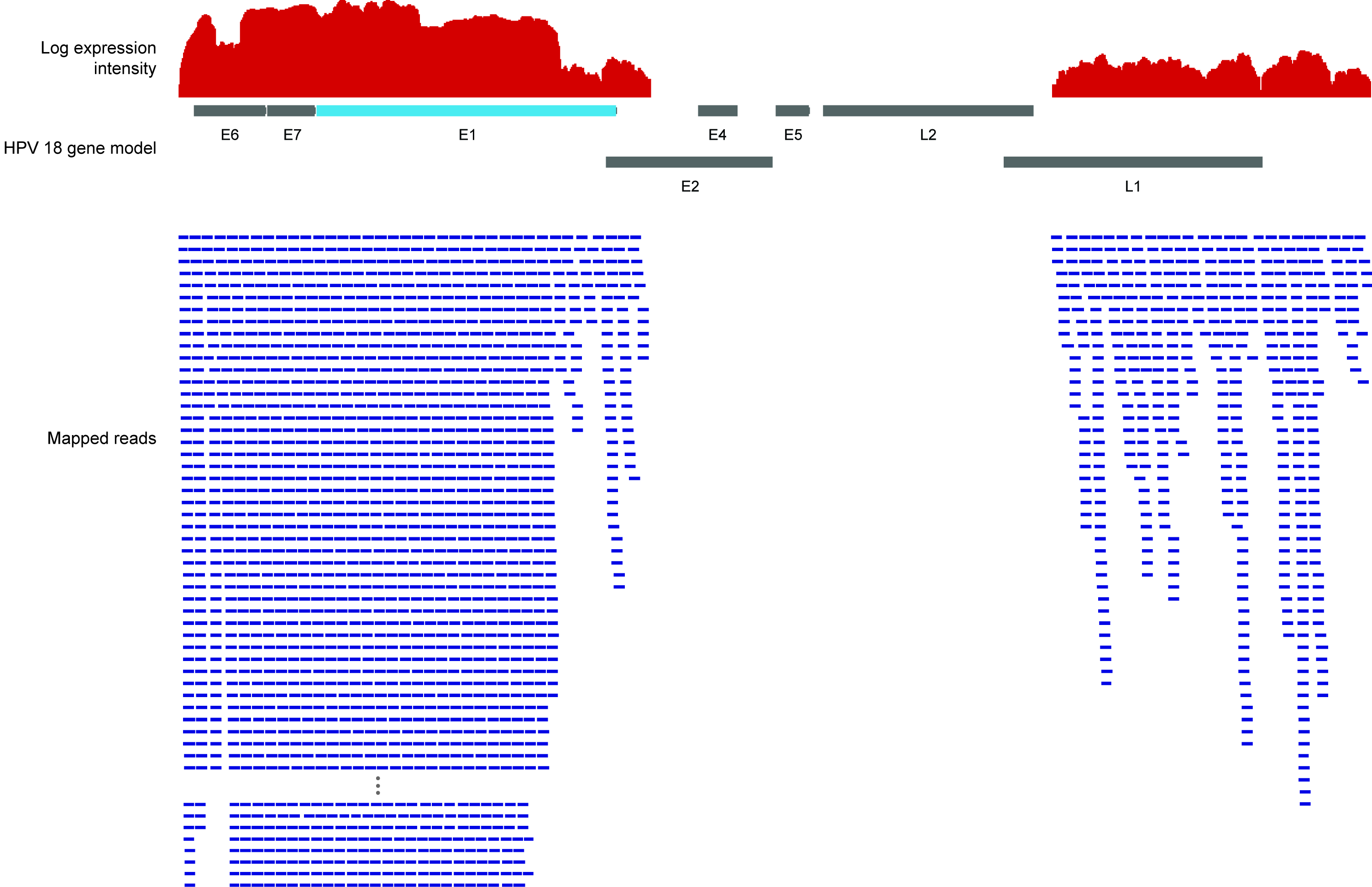


Supplementary Figure 3. **Mapping of RNA-seq reads from HeLa cells to the HPV-18 genome**. HPV-18 gene models are shown along with the RNA-seq expression intensity (log_10_ #mapped sequence tags per position; top track in red) and individual mapped sequence reads (bottom track in blue). The dominant expressed product of E1 corresponds to the previously reported E6-E7-E1 polycistron with a truncated E1 ORF [10, 12, 14]. Nevertheless, the entire E1 ORF is expressed as mRNA in HeLa cells, consistent with previous reports of a 70kD E1 protein product [11].
